# Optimized Driving Scheme for Three-Color Electrophoretic Displays Based on the Elimination of Red Ghost Images

**DOI:** 10.3390/mi15101260

**Published:** 2024-10-15

**Authors:** Mouhua Jiang, Zichuan Yi, Jiashuai Wang, Feng Li, Boyuan Lai, Liangyu Li, Li Wang, Liming Liu, Feng Chi, Guofu Zhou

**Affiliations:** 1 School of Electronic Information, University of Electronic Science and Technology of China, Zhongshan Institute, Zhongshan 528402, China; 2022024132@m.scnu.edu.cn (M.J.); 202221021120@std.uestc.edu.cn (J.W.); 202322310333@std.uestc.edu.cn (F.L.); laibo1123@163.com (B.L.); liulmxps@126.com (L.L.); chifeng@semi.ac.cn (F.C.); 2 Guangdong Provincial Key Laboratory of Optical Information Materials and Technology, South China Academy of Advanced Optoelectronics, South China Normal University, Guangzhou 510006, China; guofu.zhou@m.scnu.edu.cn; 3 School of Information Engineering, Zhongshan Polytechnic, Zhongshan 528400, China; liangyu1012@126.com (L.L.); creekxi@163.com (L.W.)

**Keywords:** electrophoretic display, driving scheme, red ghost image, flickering

## Abstract

Three-color electrophoretic display (EPD) is emerging as a display technology due to its extremely low energy consumption and excellent reflective properties. However, in the process of black and white color image transition, due to the different driving characteristics of red particles, the particles within the three-color EPD cannot be ideally driven to the target position, resulting in the appearance of a red ghost image. For this reason, this study utilized the COMSOL 5.6 finite element simulation method to construct a three-dimensional simulation model to explore the motion characteristics of electrophoretic particles, and then proposed a new driving scheme. The driving scheme aimed to drive red particles to the target position and eliminate the red ghost image by optimizing the pixel erasing stage and employing a high-frequency oscillating voltage. The final experimental results showed that after adopting the proposed driving scheme, the red ghost image was reduced by 8.57% and the brightness of the white color image was increased by 17.50%. This method effectively improved the display performance of three-color EPDs and contributed to the better application of three-color EPDs in the field of high-reflectivity and high-quality display.

## 1. Introduction

Three-color electrophoretic display (EPD) is a low-power and highly visible display technology [1,2,3]. Three-color EPDs exhibit excellent reflectivity and viewing angles compared to traditional display technologies like liquid crystal displays (LCDs) and organic light-emitting diodes (OLEDs) [4,5,6]. Additionally, they effectively address the limitations of traditional EPDs in multi-color display performance [7,8,9]. However, their refresh rates and color performance still lag behind the vibrancy and depth offered by LCDs and OLEDs, and compared with traditional EPDs, three-color EPDs have a complex spatial distribution of particles due to the increase in particle types within microcapsules, which leads to red particles with different characteristics exhibiting greater resistance to motion than black and white particles [10,11,12].

As a superior reflective display, three-color EPDs performed well in displaying static images [13,14,15]. However, due to the different characteristics of red particles, problems such as red ghost images, screen flickering, and slow response times might occur during the color transition process [16,17]. Therefore, researchers conducted a series of studies. The researchers discovered that adjusting the driving voltage sequence of charged particles in three-color EPDs, known as the driving scheme, enabled the display of different colors. Therefore, it was of great significance to improve the performance of three-color EPDs by optimizing the driving scheme. To reduce the response time, the effect of image retention time on response delay was studied by Kao et al. [18]; they showed that removing certain driving waveforms could improve the response speed of EPDs without affecting performance. A novel valveless water pump was introduced by Mao et al., utilizing electrohydrodynamic and immiscible fluid interfaces for flexible fluid driving. Additionally, KAN was employed for the predictive modeling of this flexible EHD pump, with its performance and design being innovatively optimized [19,20]. Applying multiple voltages to three-color EPDs could effectively activate electrophoretic particles, reduce the viscous resistance of particle motion, and shorten the response time, as has been shown by other studies [21,22]. Although these methods were effective in shortening the response time of three-color EPDs, high amplitude voltages could not drive red particles efficiently and inevitably led to the appearance of the red ghost image. Therefore, it was crucial to resolve the red ghost image during black and white color image transition [23,24]. A fast video half-toning algorithm was proposed by Kao et al. [15]; this algorithm solved image flickering and the ghost image problem. However, this method led to a degradation of the image display quality. A novel driving scheme was put forth by Wang et al. [25], which reduced the hysteresis phenomenon of charged particles and used multi-stage oscillation voltages to suppress the generation of the red ghost image. Nevertheless, the method exhibited a prolonged response time. Therefore, it became particularly important to design a driving scheme suitable for three-color EPDs based on the oscillation driving voltage to eliminate the red ghost image.

This study investigated causes of the red ghost image in three-color EPDs and addressed the issue of the red ghost image during display processes. A simulation model of three-color EPDs was developed, and an improved driving scheme was proposed. Compared with the traditional driving scheme, the red ghost image was suppressed and eliminated during the driving and display process.

## 2. Driving System of Electrophoretic Displays

### 2.1. Principle of Three-Color EPDs

**Figure 1:**
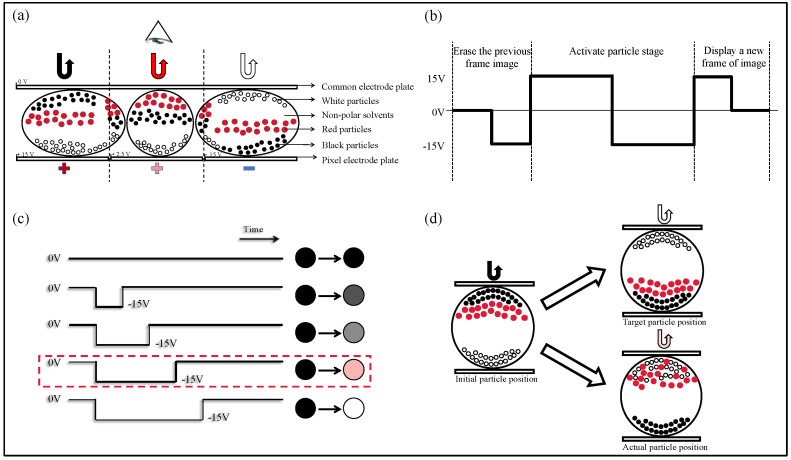
Principle of the driving scheme and the red ghost image of three-color EPDs. (**a**) Schematic diagram of three-color EPD structure; (**b**) traditional driving scheme diagram; (**c**) driving images of three-color EPD grayscale transition; (**d**) particle motion state diagram of the red ghost image.

Under the control of the driving scheme, three-color EPDs display images by reflecting ambient light through the movement of charged black, white, and red particles, as shown in Figure 1a. The structure consists of electrode plates, non-polar solvents, microcapsules, and particles of three different colors. In practical applications, the brightness, chromaticity, and stability of three-color EPDs are key indicators for evaluating the performance of the driving scheme. The slow motion of electrophoresis particles causes the driving voltage to be relatively high, as well. However, this does not affect the three-color EPD as a low-power display device. The reason is that all three particles within the three-color EPD have a bistable characteristic, and no voltage is required to maintain the display after the particles are driven. Traditionally, the driving scheme is divided into three stages; the original image erasure stage, the particle activation stage, and the new image rewriting stage, as depicted in Figure 1b. In each stage, the display is accomplished by altering the polarity of the driving voltage between two electrodes of the three-color EPD, thereby changing the position of charged particles within the microcapsule system. However, in a traditional driving scheme, the erasing stage typically involves a voltage of −15 V, and the activation stage employs a square wave with an amplitude of 30 V and a bias of 0, which results in a long driving time, noticeable flickering, and the occurrence of the red ghost image during driving processes, as illustrated in Figure 1c. When the three-color EPD transitions from a black to white color image, the mixing degree of charged particles inside three-color EPD microcapsules is great, and it is more likely to be confused than within the traditional EPD microcapsules. When red particles mix with white particles, the red ghost image may appear during color transition. The reason for this phenomenon is that the movement speed of red particles is slow, which in turn causes them to stay above the microcapsules and mix with other particles. When the target grayscale is white, the red ghost image is particularly prominent, which can also cause a decrease in the brightness when displaying the white color image, as shown in Figure 1d.

### 2.2. Design Principles of Simulation Models

**Figure 2:**
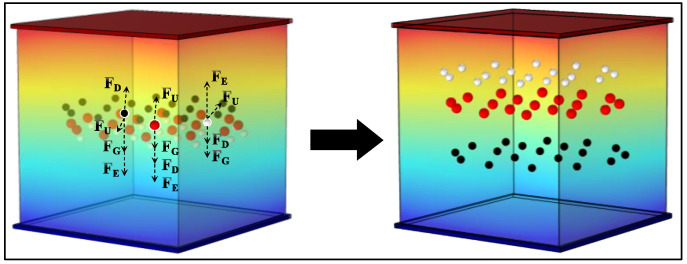
Three-color EPD simulation model diagram and force analysis.

To investigate the impact of the red ghost image on three-color EPDs and the influence of oscillating voltage on particle motion, this study employed COMSOL [26,27] to establish a particle motion model for three-color EPDs, as illustrated in Figure 2. In the constructed model, its structure consisted of electrode plates, microcapsules, and electrophoretic particles of three colors. Additionally, the three-dimensional model incorporated coupled gravity fields, fluid particle fields, and electrostatic fields. As the charged three-color particles moved within the microcapsule, they were subjected to the combined forces of particle gravity, drag from the non-polar solvent, electric field force, and inter-particle interaction forces. The comprehensive force analysis of three-color particles was displayed, elucidating the different forces acting on each particle in Figure 2. These forces adhered to Newton’s second law of motion [28,29], as expressed in Equation (1):

(1) $$F_{t}=F_{G}+F_{D}+eZE+F_{U}$$

where $F_{t}$ represents the resultant or total force acting on particles, and $F_{G}$, $F_{D}$, eZE, and $F_{U}$ denote gravity, drag force, electric field force (i.e., $F_{E}$), and interaction force, respectively. The elementary charge and the number of charges carried by particles are represented by e and Z, while the electric field strength generated by the voltage of the driving scheme is denoted as E, as shown in Equation (1). The gravitational force on charged particles and the drag force due to the non-polar solvent in the simulation model can be calculated using the Stokes equation [30,31], as indicated in Equations (2) and (3):

(2) $$F_{G}=\frac{\rho_{p}-\rho }{\rho_{p}}m_{p}g$$

(3) $$F_{D}=3\pi \mu d_{p}\left(u-v\right)$$

Here, $m_{p}$ represents the mass of the particle, $\rho_{p}$ represents the density of particles, ρ represents the density of the non-polar solvents, g represents the acceleration due to gravity (approximately 9.8 $m/s^{2}$ below sea level), μ represents the dynamic viscosity of the surrounding fluid, $d_{p}$ represents the particle size, u represents the velocity of the surrounding fluid, and v represents the velocity of particles.

In the simulation model, when the particles and surrounding fluid had the same density, their buoyancy value approached zero. In this case, particles were called suspended particles. Combining Equations (1)–(3), we obtained a simplified expression for the particle motion equation, as expressed in Equation (4).

(4) $$\frac{d^{2}q}{dt^{2}}=\frac{\rho_{p}-\rho }{\rho_{p}}g+\frac{1}{\tau_{p}}\left(u-v\right)$$

(5) $$\tau_{p}=\frac{\rho_{p}{d_{p}}^{2}}{18\mu }$$

Equation (4) introduces the constant $\tau_{p}$. $\tau_{p}$ has a time unit, which is the Lagrangian time scale or particle velocity response time, which has a significant impact on the simulation results, as shown in Equation (5). And the interaction force between particles can be calculated by Equation (6):

(6) $$F_{U}=\frac{e^{2}}{4\pi \varepsilon_{0}}\sum_{i=1}^{N}ZZ_{i}\frac{r-r_{i}}{{\left|r-r_{i}\right|}^{3}}$$

where $\varepsilon_{0}$ denotes the vacuum dielectric constant, $Z_{i}$ denotes the number of charges carried by particles, and $r-r_{i}$ denotes the distance between this particle and other particles.

**Figure 3:**
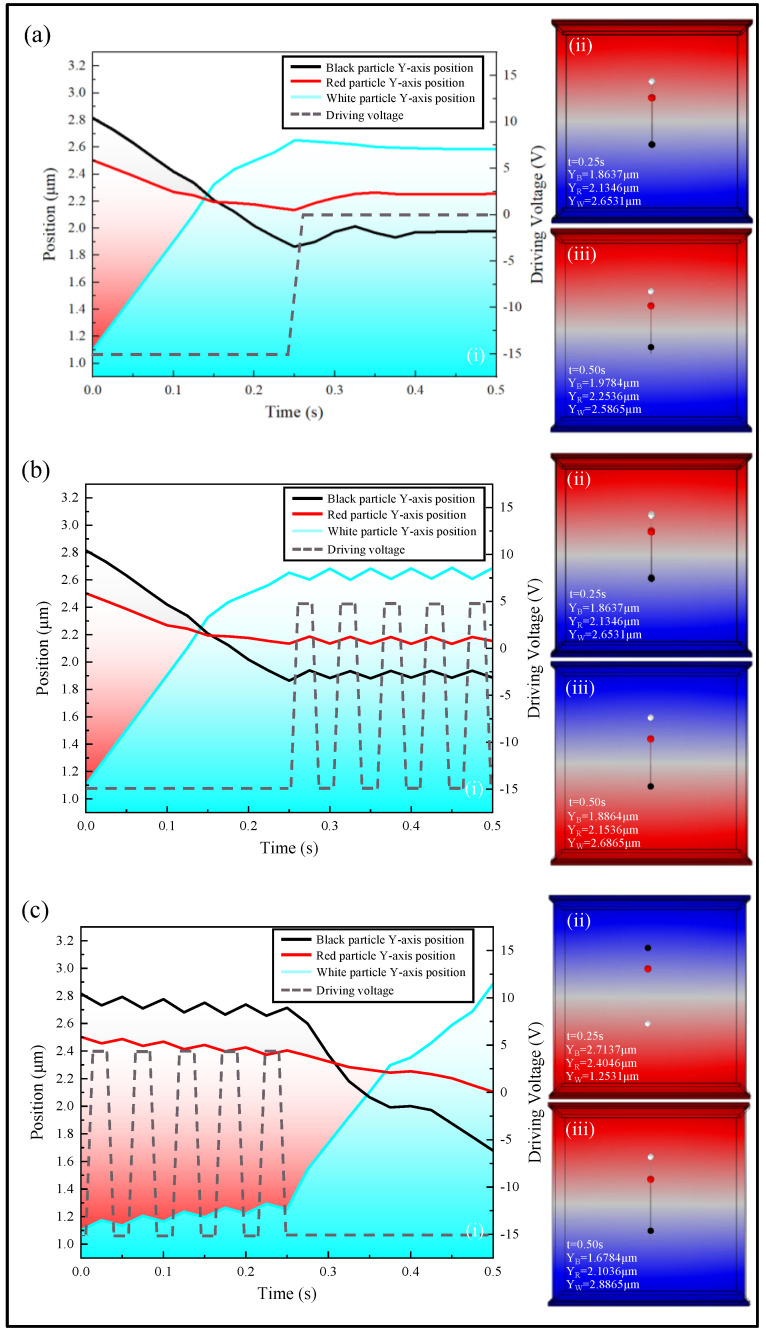
The simulated y-axis position curves of three-color EPD particle motion. (**a**) (i) showed the Y-axis position curve of three colored particles, (ii,iii) were the particle position maps at t = 0.25 s and t = 0.50 s under the traditional driving scheme; (**b**) (i) showed the Y-axis position curve of three colored particles, (ii,iii) were the particle position maps at t = 0.25 s and t = 0.50 s under the oscillation driving voltage proposed in this paper; (**c**) (i) showed the Y-axis position curve of three colored particles, (ii,iii) were the particle position maps at t = 0.25 s and t = 0.50 s under the oscillation driving voltage applied in advance.

As shown in Figure 3, we have illustrated the vertical motion trajectories of three colored particles under different driving voltages. Figure 3a presents the vertical motion trajectories of particles in a three-color EPD driven directly by the traditional scheme. When the three-color EPD transitions from a black to white color image, the velocity of three color particles also changes due to the change in the resultant force acting on particles. When the driving voltage reached zero at 0.25 s, there was no electric field force between the two electrode plates. Under the influence of the interaction force between particles, the three colored particles underwent varying degrees of displacement, moving from the position in Figure 3a(ii) to the position in Figure 3a(iii). This led to a disordered particle distribution, which increased the likelihood of the red ghost image.

In contrast, as shown in Figure 3b, the particle motion trajectory remained consistent with that in Figure 3a before 0.25 s. However, after 0.25 s, an oscillation driving voltage was introduced, causing the three colored particles to move from position (ii) to position (iii) in Figure 3b. Compared to the previous case, this oscillation driving voltage adjusted the positions of the three colored particles, increasing the movement distance of the three colored particles slightly relative to Figure 3a. This separation of red and white particles stabilized particle positions, enabling the precise control of the grayscale display on the three-color EPD. As shown in Figure 3c, an oscillation driving voltage was applied when the particle was stationary, and the three colored particles acquired an initial velocity. Therefore, compared to Figure 3b, these particles achieved a greater travel distance within the same driving time, but this was not controllable and could result in inconsistent brightness when the white color was displayed. The simulation results showed that the introduction of an oscillation driving voltage could stabilize the positions of the three colored particles, allowing precise color control. The accuracy of these simulation results would be further validated by actual driving experiments on three-color EPDs.

## 3. Experimental Results and Discussion

### 3.1. Experimental Platform

To test the feasibility of the driving scheme, an optical experimental setup was built by us in early research efforts [32]. This experimental setup helped to test the brightness level and red saturation of three-color EPDs, and also characterized the display quality when a white color was displayed based on the red saturation and brightness level. The test object was the three-color EPD DEP0273, and ten devices of the same type were used to repeat the experiment. And the experimental setup included a computer, function generator, signal amplifier, and colorimeter. This experimental setup was used to test the traditional driving scheme and the proposed driving scheme.

### 3.2. Design and Optimization of Driving Schemes

**Figure 4:**
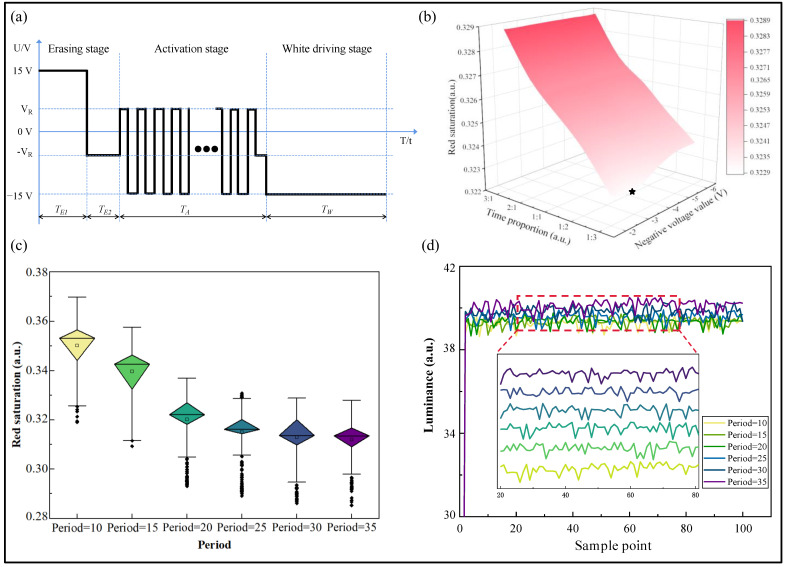
The driving scheme and optimization results of this study. (**a**) The driving scheme proposed in this paper; (**b**) red saturation varying with parameters of the erasing stage; (**c**) the relationship between red saturation and activation period; (**d**) the relationship between brightness and activation period when a white-colored image was displayed.

In this paper, a new driving scheme was proposed through the simulation results derived from the simulation model, which was optimized for the erasing stage and activation stage, as shown in Figure 4a. During the erasing stage, a positive voltage with a duration of $T_{E1}$ and a negative voltage with a duration of $T_{E2}$ were used in this driving scheme; their amplitudes were 15 V and $-V_{R}$ V, respectively. In the activation stage, an oscillating voltage with a duration of $T_{A}$ was used by the proposed driving scheme, and the amplitude of the oscillating voltage was $\left|V_{R}+15\right|$ V, where $V_{R}$ was the driving voltage for red particles. Finally, in the white-colored image driving stage, a negative voltage with a duration of $T_{W}$ was used by the proposed driving scheme, with an amplitude of −15 V.

To further optimize the proposed driving scheme, all parameters must be tested to evaluate their impact on the elimination of the red ghost image, aiming to achieve optimal performance for the driving scheme. During the erasing stage, $-V_{R}$, $T_{E1}$, and $T_{E2}$ need to be investigated. Since the driving voltage threshold for red particles was significantly lower than that for black particles, $-V_{R}$ was set to −2 V, −3 V, −4 V, −5 V, and −6 V, and the duration ratio $T_{E1}:T_{E2}$ was set to 3:1, 2:1, 1:1, 1:2, and 1:3. Since the red ghost image had a large effect on the transition of the three-color EPD from a black to white color image, then the experiment was carried out under conditions where the original image of the three-color EPD was set to black and the target image was set to white, and ten three-color EPDs DEP0273 were tested, and the results were averaged. Figure 4b shows the saturation of the red ghost image when a white color is displayed. It could be observed that, at the same $-V_{R}$, the red saturation reached the highest value when the ratio $T_{E1}:T_{E2}$ was maximized. This indicated that, in the absence of a sufficient red erasing stage, red particles could not be adequately separated from the top of the microcapsule. As the proportion of the red erasing stage increased, the red saturation decreased. This phenomenon suggested that increasing the duration of the red erasing stage could enlarge the distance between red particles and the top of the microcapsule, thereby reducing residual red particles at the top of the microcapsule. When the ratio $T_{E1}:T_{E2}$ was certain, the lowest red saturation was observed for $-V_{R}$ of −3 V; this confirmed the optimal value of $-V_{R}$, as indicated by the star-marked point in Figure 4b, which was favorable for the red particles to move rapidly toward the bottom of the microcapsule. Therefore, $-V_{R}$ was set to −3 V and the ratio $T_{E1}:T_{E2}$ was set to 1:3, which minimized the effect of the red ghost image.

In the activation stage of the proposed driving scheme, the optimized oscillation voltage was employed. Since the human eye could detect flickering when the oscillation frequency was below 25 Hz, the activation period was set to less than 40 ms to avoid flickering during optimization. To determine optimal parameters for the activation stage, the activation period was set to 10 ms, 15 ms, 20 ms, 25 ms, 30 ms, and 35 ms; the experimental results are shown in Figure 4c,d. It was evident that, during the white color image display stage, the variance of the red saturation data decreases to a minimum and the lowest red saturation occurred when the activation period was set to 35 ms. Additionally, with an activation period of 35 ms, higher stability was achieved when the white color was displayed. The maximum brightness was maintained. Therefore, the parameters set under these conditions were most effective in eliminating the red ghost image.

### 3.3. Display Performance Comparison of Driving Schemes

To validate the effectiveness of the proposed driving scheme in eliminating the red ghost image, a traditional driving scheme [33] was used for performance comparison, and the three-color EPD was driven by both schemes at the same time. For the traditional driving scheme, the erasing stage duration was set to 500 ms, the activation period to 200 ms with four activations, and the black and white driving stage to 500 ms.

**Figure 5:**
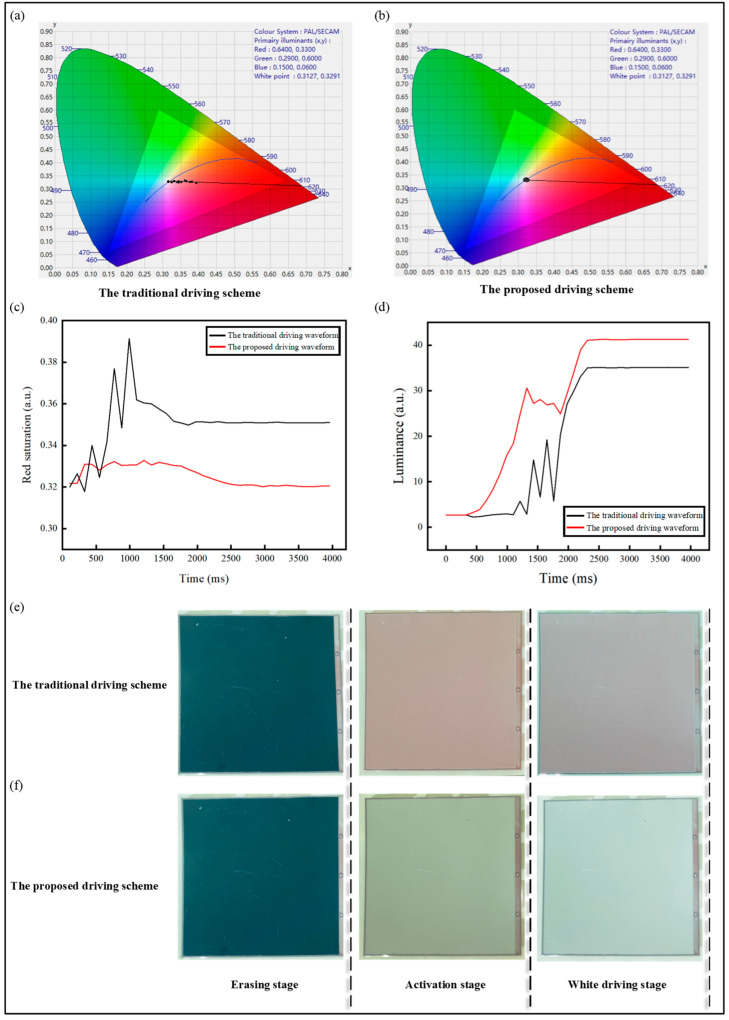
Driving variation of three-color EPDs under two driving schemes. (**a**) The chromaticity changes in the traditional driving scheme [17,22,33]; (**b**) the chromaticity changes in the proposed driving scheme; (**c**) diagram of red saturation during color transition; (**d**) diagram of brightness during color transition; (**e**) the driving process diagram of the traditional driving scheme; (**f**) the driving process diagram of the proposed driving scheme.

The CIE Yxy chromaticity diagrams of three-color EPDs driven by the traditional and proposed driving schemes are shown in Figure 5a,b, respectively, where changes in red saturation could be observed. It was evident that under the traditional driving scheme, red saturation exhibited significant fluctuations during the driving process, reaching a peak of 0.39. Residual red particles also compromised the accuracy of the white color image, resulting in a red ghost image with a final red saturation value of 0.35. In contrast, the proposed driving scheme demonstrated only minor oscillations in red saturation during the driving process, indicating that the red erasing and activation stage effectively removed red particles, which prevented the occurrence of the red ghost image. The final red saturation value in the white color image was 0.32, which fell within the normal range of the white region in the chromaticity diagram.

Figure 5c showed the temporal variation curve of the red saturation value of the image under two driving schemes. Therefore, compared to the traditional driving scheme, the proposed driving scheme reduced the red ghost image of the white-colored image by 8.57%.

At the same time, the red ghost image affected the brightness of the three-color EPD. As shown in Figure 5d, variation curves of brightness over time under two driving schemes were presented. When the white color was displayed, the brightness of the three-color EPD reached 41.29 under the proposed driving scheme and 35.14 under the traditional driving scheme, with an increase in brightness of 17.50%.

Figure 5e,f presents the image color transition of the three-color EPD under two driving schemes. The image display behavior of the three-color EPD under the traditional driving scheme is shown in Figure 5e. It can be observed that the red ghost image appeared during the activation stage, with a noticeably high level of red saturation at this stage. Ultimately, the final white-colored image clearly shows the presence of the red ghost image. From Figure 5f, it can be seen that the red ghost image did not appear during the image display process, and the image maintained low red saturation consistently throughout the driving process. Furthermore, after the final driving stage, the white-colored image was displayed accurately. Therefore, this further validated the reliability of the simulation model and the proposed driving scheme.

## 4. Conclusions

In this paper, a three-dimensional simulation model for the electrophoretic particle motion of the three-color EPD was presented, and a novel driving scheme was proposed to improve the performance of the three-color EPD based on our model results. Compared with another driving scheme, the proposed driving scheme could effectively eliminate the red ghost image and obtain a stable white-colored image display, which improved the display performance of EPDs. Therefore, our proposed driving scheme can provide some reference value for the application and development of three-color EPDs. The optimized three-color EPD can be applied in medical and educational devices to meet the requirements of low energy consumption and high readability. In addition, because the three-color EPD belongs to the field of microfluidics, our research results also provide a certain reference value for driving microfluid.

## Data Availability

Data are contained within the article.
